# Observations of the Gas Phase Composition of the 2024 BioLab Industrial Plume in the Atlanta Metropolitan Area

**DOI:** 10.1021/acs.estlett.6c00160

**Published:** 2026-03-26

**Authors:** Christine A. Harper, Linda Arterburn, Katherine Paredero, Rime El Asmar, Mariama L. Stewart, David J. Tanner, James M. Roberts, John J. Orlando, Joseph Sadighi, Rodney J. Weber, L. Gregory Huey

**Affiliations:** † School of Earth and Atmospheric Sciences, 1372 Georgia Institute of Technology, Atlanta, Georgia 30309, United States; ‡ Atmospheric Chemistry Observations & Modeling Laboratory, 116511 NSF National Center for Atmospheric Research, Boulder, Colorado 80301, United States; § School of Chemistry & Biochemistry, Georgia Institute of Technology, Atlanta, Georgia 30309, United States

**Keywords:** Atmospheric chemistry, Chemical plume, Pool chemicals, Halogens, HNCO, Public health, Chemical ionization mass spectrometry

## Abstract

A fire at a pool chemical manufacturing facility in Conyers, Georgia, on September 29, 2024, released a persistent chemical plume that impacted the Atlanta metropolitan area, causing evacuation of more than 17,000 people. We used chemical ionization mass spectrometry (HR-TOF-CIMS) in Midtown Atlanta (21 miles from the facility), and in Conyers (quadrupole CIMS) to characterize plume composition. We observed unexpectedly high concentrations of Br_2_ (up to 1.4 × 10^2^ ppbv) dominating early plumes, along with elevated levels of HNCO (31 ppbv) and numerous other compounds. Twenty-six species were identified, including reactive nitrogen-containing compounds (e.g., HNCO, cyanoacetic acid, and cyanamide) and oxygenated volatile organic compounds (e.g., acetaldehyde). Br_2_ concentrations in Midtown exceeded United States Environmental Protection Agency (EPA) 1 h Acute Exposure Guideline Levels (AEGL-1) thresholds by a factor of 4. Later measurements in Conyers showed Cl_2_ reaching 3.7 × 10^2^ ppbv during the second week. Given that Midtown observations were 21 miles downwind, concentrations near the source in Conyers were likely 2 orders of magnitude higher. This is the first comprehensive chemical characterization of a pool chemical decomposition plume revealing complexity beyond simple halogen release and highlighting health risks from simultaneous exposure to multiple respiratory irritants.

## Graphical abstract

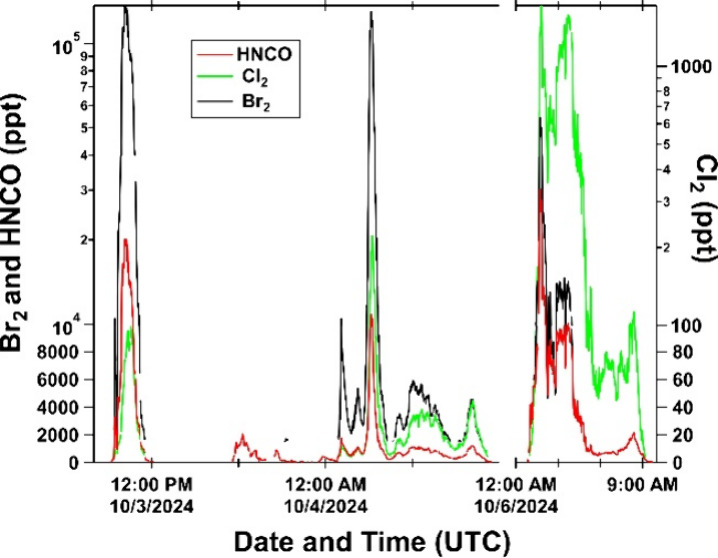

## Introduction

A fire at a pool and spa chemical manufacturer (BioLab) in Conyers, Georgia, on September 29, 2024, initiated a chemical plume impacting the Atlanta metropolitan area for over 2 weeks. The facility, located 21 miles east of Midtown Atlanta (Figure S1), contained over 5,000,000 kg of chlorinating and brominating agents.^1^ The initial fire was contained but water exposure caused unwanted reactions producing a persistent plume, leading to an evacuation of more than 17,000 people and shelter-in-place orders.^1^ Easterly winds (Figure S2) transported the plume into densely populated areas during the first week of October.

Initial public health announcements emphasized possible exposure to Cl_2_ and HCl, with Br_2_ mentioned to a lesser extent.^2–4^ However, the chemistry of pool chemicals is complex as these products use cyanuric acid (CYA) derivatives as chlorine and bromine sources (Figure S3).^1,5^ The primary compounds stored at BioLab^1–3^ were sodium dichloroisocyanurate (DCCA), trichloroisocyanuric acid (TCCA),^5^ and bromochloro-5,5-dimethylimidazolidine-2,4-dione (BCDMH).^6^

From 2004 to 2024, six major pool chemical facility incidents occurred in Texas, Louisiana, Massachusetts, and Georgia, resulting in evacuations of nearby residents, yet comprehensive measurements of plume composition were not reported.^7–13^ These incidents are a recurring public health concern, and the decomposition of cyanuric acid derivatives can produce reactive halogenated organics, nitrogen-containing compounds, and other hazardous species whose identities, concentrations, and health impacts are poorly understood.^1,5,6^ The BioLab incident provided a unique opportunity to characterize emissions from prolonged chemical decomposition following a major pool chemical facility fire affecting a densely populated metropolitan area. We measured plume composition in Midtown Atlanta with a high-resolution time-of-flight chemical ionization mass spectrometer (HR-TOF-CIMS) and a quadrupole CIMS in Conyers.

## Materials and Methods

The HR-TOF-CIMS measured ambient air in Midtown Atlanta from an inlet mounted on the roof of the Ford Environmental Science and Technology Building at Georgia Tech (33.7784° N, 84.3962° W) from September 30 to October 9, 2024. A Georgia Tech mobile laboratory (GTML) with air monitoring equipment^14^ and a quadrupole CIMS^15^ was deployed at Conyers City Hall (33.6733° N, 84.0195° W) from October 3 to October 15, 2024.

At the time of the incident, the HR-TOF-CIMS (Aerodyne Inc.) was configured as a thermal decomposition CIMS using I^–^ chemistry to perform postmission calibrations of acetyl peroxynitrates (PANs).^16,17^ This configuration also enabled the measurement of many other species including halogens, nitrogen containing compounds, and potentially oxygenated volatile organic compounds (OVOC).^18–22^ Two upgrades from Lee et al.^23^ included (1) a TOF analyzer replacing the quadrupole mass filter (resolution ∼ 5000 m/Δm) and (2) a vacuum ultraviolet lamp ion source (Heraeus PKS 106).^24^

In Midtown, air was sampled at 3 slpm (standard liter per minute) through a 5 m long x 8 mm ID Teflon inlet tube connected to the TOF-CIMS which was calibrated continuously with ^13^C-labeled PAN.^23^ Cl_2_ and Br_2_ were calibrated using permeation tubes (Vici Metronics).^25^ HNCO was produced from CYA thermal decomposition and quantified by conversion to NO.^20^ HCN, ethylene oxide, acetaldehyde and methyl isocyanate were calibrated using high pressure standards. Thirteen species were quantified by their vapor pressures (Table S1). Sensitivities for seven species were estimated from previous work.^26–28^ The instruments deployed in the GTML^14^ in Conyers are a Tapered Element Oscillating Microbalance (TEOM) ambient particulate monitor,^14^ a Purple Air aerosol monitor,^29^ a whole air filter sampler (Table S2),^30^ and a quadrupole CIMS.^25^ All CIMS measurements are reported as 1 min averages, with TEOM and Purple Air data averaged for 1 min and 2 min, respectively. The methods employed could not detect HCl or other important gaseous species, such as cyanogen chloride (ClCN),^27^ and did not provide a detailed analysis of aerosol phase chemical composition.

## Results

### Midtown

We began measurements with the HR-TOF-CIMS in Midtown Atlanta on September 30, 2024. On the evening of October 3, we observed unexpectedly high concentrations of Br_2_ and HNCO (up to 1.4 × 10^2^ and 31 ppbv, respectively) with lower levels of Cl_2_. We used HNCO as a plume tracer because it is a CYA thermal decomposition product, background levels in Atlanta are <1 ppbv (Figure S4), and it is photochemically stable allowing for daytime detection.^18,23,31^ Using HNCO, meteorological observations, and NOAA HYSPLIT back trajectories (Figures S5–S9), we identified four plume interceptions (three nocturnal and 1 daytime) in Midtown Atlanta between October 3 and 6, 2024. The daytime plume (October 3 18:00 UTC) was identified by elevated HNCO and southeasterly winds. All concentrations reported represent enhancements above background levels. Figure 1 shows the time series and nocturnal plume correlations of Br_2_, Cl_2_, and HNCO in Midtown.

**Figure 1:**
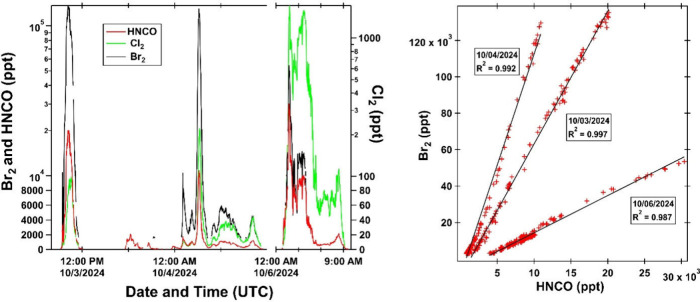
(Left) Time series of Br_2_ (black, left axis), Cl_2_ (green, right axis), HNCO (red, left axis) mixing ratios observed in Midtown Atlanta. Note the left axis is linear below 10^4^ pptv and logarithmic above and the right axis is linear below 100 pptv and logarithmic above. (Right) Correlations between Br_2_ and HNCO concentrations during nocturnal plume interceptions. R^2^ for the plume interceptions on 10/03, 10/04, and 10/06 are 0.997, 0.992, and 0.987. From October 3 to 6, the Cl_2_/Br_2_ ratio increases by more than a factor of 200. In particular, on 10/06 from 01:47 to 04:03 UTC, this ratio increases by more than a factor of 5. After this, Br_2_ concentrations are below the limit of detection (∼1.5 ppbv).

Twenty-six I^–^ cluster ions (e.g., I^–^(HNCO)) were identified as having elevated signal levels in the BioLab plumes impacting Midtown Atlanta. We obtained calibrations for 17 of these species. Species (e.g., Br_2_, Cl_2_, HCN, and HNCO) were identified based on distinct isotopic signatures (Figures S7–S8), mass defects, known chemistry, and unambiguous molecular formulas. These species are listed in bold in Table 1 along with peak 1 min average concentrations and estimated calibration uncertainties calculated from the combined uncertainties in vapor pressure, mixing ratios, and gas flows. For the molecular formula C_2_H_4_O which is more ambiguous, mixing ratios and uncertainties are based on calibration data for acetaldehyde. Nine species were not calibrated due to complex isomeric possibilities, unavailability of calibration sources, or low signal levels (Table S3).

**Table 1:** Midtown Detected Species Concentrations and Uncertainties

Species	Highest Observed Concentration (pptv)	Estimated Uncertainty for Calibrated Species (%)
C_2_H_4_O (ethylene oxide^b^, **acetaldehyde** ^b^ **,** vinyl alcohol)	1.4 × 10^6^	27
Br_2_ (**bromine** ^c^)	140 × 10^3^	27
HNCO (**isocyanic acid** ^a^, cyanic acid, fulminic acid)	30 × 10^3^	16
Cl_2_ (**chlorine** ^c^)	1.7 × 10^3^	30
C_3_H_3_NO_2_ (**cyanoacetic acid** ^c^, HOCH_2_–C(=O)CN)	1.6 × 10^3^	27
CH_4_N_2_O (**urea** ^a^, ammonium cyanate)	870	27
ClNO_2_ (**nitryl chloride** ^d^, nitrosyl hypochlorite)	310	36
HCN (**hydrogen cyanide** ^b^, hydrogen isocyanide)	140	14
BrCl (**bromine chloride**)^e^	85	28
Cl_3_N (**trichloroamin**e)^f^	42	43
Cl_2_NH (**dichloroamine**)^f^	36	43
CH_2_N_2_ (**cyanamide**, carbodiimide, diazomethane, isocyanamide, nitrilimine)^a^	28	27
BrNO_2_ (bromine nitrite, **nitryl bromide**)^e^	6.5	36
HOBr (**hypobromous acid**)^h^	6.3	25
BrO (**bromine monoxide**)^d^	5.9	25
BrCN (**cyanogen bromide** ^d^, bromine isocyanide)	1.9	25
C_2_H_3_NO (methylisocyanate^b^, **hydroxyacetonitrile** ^g^)	1.1	>15

a Calibrated by vapor pressure.

b Calibrated with a high pressure gas standard.

c Calibrated with a permeation tube.

d Estimated from Roberts et al.^27^

e Estimated as average of Br_2_ and Cl_2_ sensitivity

f Estimated from Angelucci et al.^32^

g Estimated from Finewax et al.^33^

h Estimated from Liao et al.^28^

### Conyers

BioLab plumes impacted Conyers City Hall repeatedly after October 6, 2024. Plumes were identified by Cl_2_, as HNCO could not be quantified by the quadrupole CIMS. Cl_2_ levels above 1 ppbv were observed on seven separate occasions, with a maximum 1 min average of 3.7 × 10^2^ ppbv on the afternoon of October 7. Sustained plumes (Cl_2_ > 1 ppbv for longer than 1 h) were observed at nighttime on October 7, 12, and 13, with maximum concentrations decreasing by approximately an order of magnitude over this period (Figure S12). Br_2_ levels above the CIMS detection limit (∼100 pptv) were not observed in Conyers. High PM_2.5_ levels (>80 μg/m^3^) were observed by aerosol instruments (Figure S13) coincident with the October 7 Cl_2_ plume.

## Discussion

Midtown Atlanta experienced elevated concentrations of halogenated species, nitrogen-containing compounds, and oxygenates during the BioLab plumes. Additional species may have been present but undetected due to chemical transformations during transport or I^–^ CIMS detection limitations.

### Halogenated Species

Early plumes in Midtown Atlanta (October 3–6) contained high Br_2_ with comparatively low Cl_2_, while plumes measured in Conyers the following day (October 7) showed high Cl_2_ with Br_2_ below detection limit of the quad CIMS (∼100 pptv). The Cl_2_/Br_2_ ratio increased by more than a factor of 100 from October 3 through October 6, including a factor of 5 increase within 2.5 h on October 6 (Figure 1), after which Br_2_ fell below the TOF-CIMS detection limit. In solution, HOCl readily oxidizes Br^–^ to form HOBr, which subsequently forms Br_2_ through acid-catalyzed disproportionation.^6^ The rate of HOCl + Br^–^ is pH-dependent, with acidic conditions favoring faster Br^–^ consumption.^34^ However, quantitative modeling is not possible given the unknown and likely changing solution pH at the active demolition site. As Br^–^ was depleted, the system likely shifted to favor Cl_2_ release, consistent with the increasing Cl_2_/Br_2_ ratio observed across successive plumes (Figure 1). Laboratory tests (SI) show Cl_2_ can displace Br_2_ from instrument surfaces, introducing a positive Br_2_ artifact. However, in the worst case the associated Cl_2_ loss is less than 20% and too small to account for the observed Br_2_ levels. The elevated TOF-CIMS Br_2_ background and detection limit (∼1.5 ppbv) were due to recent PAN calibrations using a Br_2_ permeation tube,^35^ but were too small to impact the observed Br_2_ levels. Both effects would lead to underestimation of the Cl_2_/Br_2_ ratio, and neither can explain the rapid, structured variation in the Br_2_/Cl_2_ ratio observed across distinct plume events. We therefore conclude that the rapid loss of Br_2_ between October 6 and 7 reflects depletion of available bromine at the BioLab facility.

We also observed elevated concentrations of BrCl, BrO, BrCN, BrNO_2_, and ClNO_2_ at less than 1 ppbv. ClNO_2_, BrO, and BrCN occur naturally at similar levels.^23,24,27^ BrNO_2_ is likely formed from N_2_O_5_, analogous to ClNO_2_.^23^ The observed BrCl level is one of the highest reported^36–40^ and likely due to the extreme levels of Br_2_ and Cl_2_. Chloramines (NCl_3_, NHCl_2_), known pool products, were observed at low levels.^33,41,42^ Several chlorinated and fluorinated organics were detected but not calibrated due to isomeric ambiguity and lack of available standards. However, for the fluorinated species at least, we are likely very sensitive, and the observed levels are probably below 1 ppbv.

### Nitrogen-Containing Species

The observed nitrogen-containing species were dominated by nitrile and to a lesser extent amine functional groups. HNCO, observed at the highest levels, is a thermal decomposition product of CYA.^20^ Calibrations demonstrated HNCO formation while heating CYA to 220 °C. CYA derivative decomposition^1^ also likely explains the observed urea.^20,25^ The molecular formula CH_2_N_2_ is assigned as cyanamide, a stable nitrile isomer,^43^ observed in the thermal decomposition of CYA.^31^

For C_2_H_3_NO, one possibility is methyl isocyanate, a very toxic substance. However, we could not detect methyl isocyanate from our calibration source indicating very low sensitivity. Finewax^33^ observed similar results for methyl isocyanate and demonstrated high sensitivity with I^–^ CIMS to the isomeric species hydroxyacetonitrile (HOCH_2_CN). Therefore, we assign the observed compound as hydroxyacetonitrile.

C_3_H_3_NO_2_ is assigned as cyanoacetic acid, a stable nitrile used to calibrate the CIMS. The isomeric species HOCH_2_–C­(O)­CN has not been reported as stable. Two other nitrogen-containing species, C_2_H_5_N and C_2_HNO_3_, were observed at low signal levels.

### OVOC

Several oxygenated volatile organic compounds (OVOC) were detected. C_2_H_4_O was observed at relatively high signal levels and is tentatively assigned as acetaldehyde. We are moderately sensitive to acetaldehyde, and it is a known product of combustion and thermal decomposition processes.^44,45^ In contrast, ethylene oxide (also C_2_H_4_O) could not be detected by the CIMS from a commercial standard. However, the observed C_2_H_4_O may be from the decomposition of another oxygenate.

Additional masses corresponding to oxygenated species were detected, including C_2_H_2_O_2_, C_2_H_2_O, and C_8_H_14_O_3_. C_2_H_2_O_2_ could represent glyoxal but is highly unlikely as the I^–^-CIMS is very insensitive to this compound.^46^ C_2_H_2_O is tentatively assigned as ketene, though all isomers with this formula (ketene, ethynol, oxirene) are highly unstable.^47^ The detection of C_8_H_14_O_3_ at moderate signal levels also suggests the presence of larger oxygenated organics in the plume.

OVOC identification is complicated by potential fragmentation in the heated inlet and ionization region, which may produce smaller C_*x*_H_γ_O_γ_ masses from larger parent molecules. Without successful calibrations, concentrations and structures remain uncertain, suggesting additional unidentified oxygenated species were likely present.

### Public Health Implications

The chemically complex BioLab plumes exposed millions in metropolitan Atlanta to numerous toxic compounds.

In Midtown Atlanta, peak 1 min average concentrations of Br_2_, HNCO, and Cl_2_ reached 1.4 × 10^2^, 31, and 1.7 ppbv, respectively. EPA Acute Exposure Guideline Levels define thresholds for health effects: AEGL-1 levels (500 ppbv Cl_2_, 33 ppbv Br_2_ for 1 h) indicate noticeable discomfort, while AEGL-2 levels (2000 ppbv Cl_2_, 240 ppbv Br_2_) indicate potential irreversible health effects.^48,49^ Midtown Br_2_ exceeded the 1 h AEGL-1 threshold by a factor of 4, while Cl_2_ remained below AEGL-1. No EPA AEGL levels exist for HNCO; however, prior studies suggest exposure above 1 ppbv may cause adverse health effects.^50^ Observed HNCO concentrations in Midtown exceeded this threshold by a factor of 30.

In Conyers, Cl_2_ reached 370 ppbv, approaching AEGL-1 levels more than a week after the incident. Br_2_ was below detection limits at Conyers after October 6. However, given the high concentrations observed 21 miles downwind in Midtown, concentrations near the source in the first few days likely exceeded AEGL-2 thresholds for multiple species. BrCl also likely exceeded exposure thresholds (AEGL-2 of 870 pptv)^51^ closer to the source.

Many observed species (Table S4) have potentially adverse health effects. Combined exposure to multiple compounds could amplify health effects.^52^ These findings highlight the chemical complexity and significant public health impacts of such industrial incidents.

This study provides the first comprehensive chemical characterization of a pool chemical facility fire, revealing complexity far beyond that emphasized in public health communications. Unexpectedly, Br_2_ dominated early plume composition despite the facility containing more chlorinated compounds, with a temporal shift to Cl_2_-dominated emissions reflecting progressive Br^–^ depletion. The plumes contained elevated levels of reactive nitrogen compounds (HNCO, cyanoacetic acid, cyanamide) and oxygenates (acetaldehyde and/or larger OVOC). Simultaneous exposure to multiple respiratory irritants likely enhanced health risks. Measurements 21 miles downwind in Midtown Atlanta demonstrated concentrations exceeding health thresholds; levels in Conyers during initial releases were likely more than 2 orders of magnitude higher, based on NOAA HYSPLIT dispersion models (Figure S14), potentially reaching ppmv levels for multiple toxic species.

The species reported here represent only those detectable by I^–^ CIMS. Many other hazardous compounds may have been present but undetected due to instrumental limitations or chemical transformations during transport. PM_2.5_ concentrations were also elevated during some events, adding to health concerns, though limited PM chemical speciation limits assessment of aerosol toxicity (Table S2). This work underscores the need for improved risk assessment frameworks for pool chemical storage facilities and more comprehensive air monitoring capabilities during similar incidents. These findings provide essential baseline data for understanding public health impacts of pool chemical decomposition.

## Supplementary Material



## Data Availability

The data supporting this study can be found in the Georgia Institute of Technology Digital Repository at https://doi.org/10.35090/gatech/79956 and https://doi.org/10.35090/gatech/80593.

## Notes

Further experimental details, including calibration information, instrumentation setup at field sites, and additional figures regarding Conyers results (PDF)
