# Supplementary material for: Observations of the Gas Phase Composition of the 2024 BioLab Industrial Plume in the Atlanta Metropolitan Area
Source: Environ Sci Technol Lett. 2026 Mar 26;13(5):664–9. doi: 10.1021/acs.estlett.6c00160 (PMC13173647; doi:10.1021/acs.estlett.6c00160)
Supplement: Supplementary file 1 [file ez6c00160_si_001.pdf]

Observations of the Gas Phase Composition of the 2024 BioLab Industrial Plume in the Atlanta Metropolitan Area: Supplementary Information

Christine A. Harper[1], Linda Arterburn[1], Katherine Paredero[1], Rime El Asmar[1], Mariama L. Stewart[1], David J. Tanner[1], James M. Roberts[1], John J. Orlando[2], Joseph Sadighi[3], Rodney J. Weber[1], L. Gregory Huey[1*]

[1]School of Earth and Atmospheric Sciences, Georgia Institute of Technology, Atlanta, 30309, GA, USA
[2]Atmospheric Chemistry Observations & Modeling Laboratory, NSF National Center for Atmospheric Research, Boulder, 80301, CO, USA
[3]School of Chemistry & Biochemistry, Georgia Institute of Technology, Atlanta, 30309, GA, USA

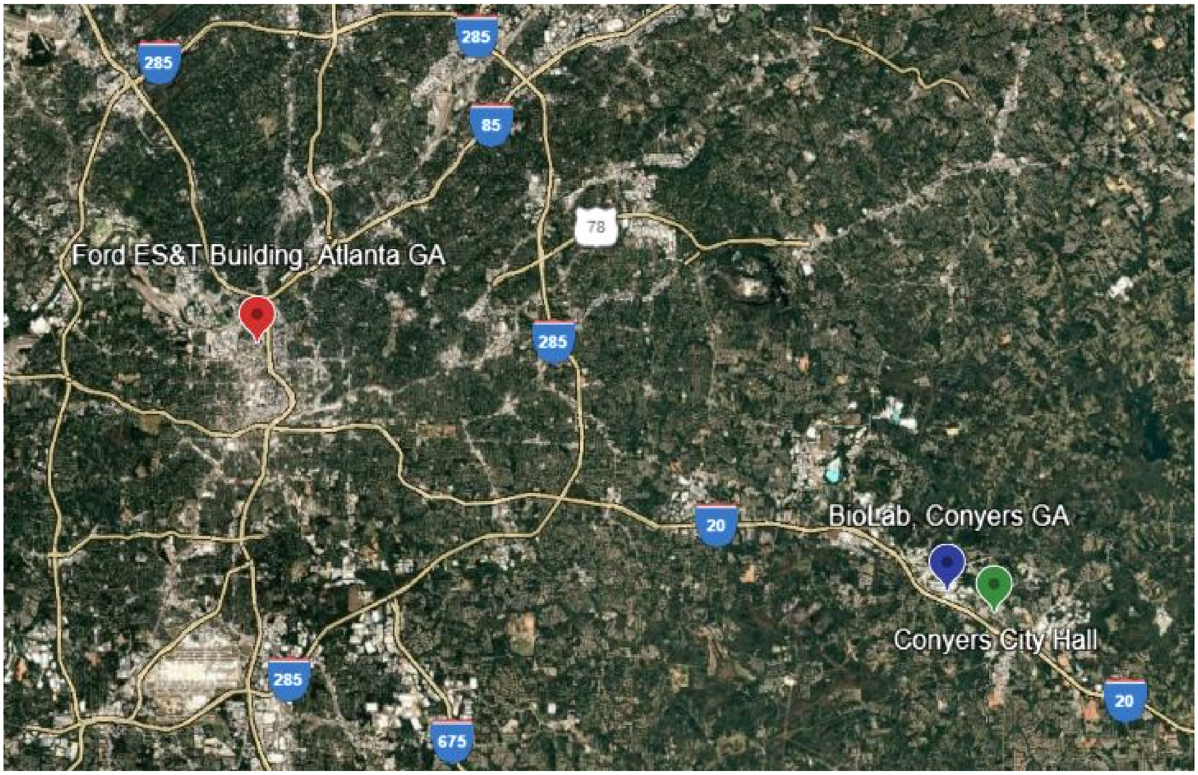

Figure S1. The map above shows the three locations of interest. The Ford ES&T building is marked in red; Conyers city hall is marked in green, and the BioLab manufacturer is marked in blue.

Meteorological Conditions

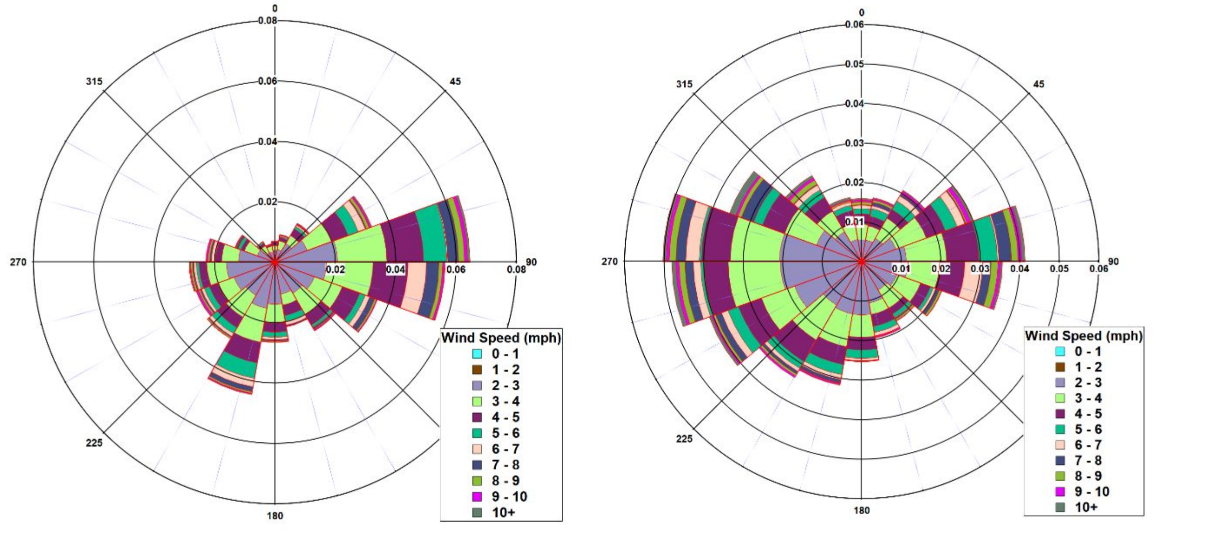

Figure S2. The left wind rose shows the wind direction in Midtown Atlanta; color coded to wind speed in miles per hour from September 30th to October 6th. The right wind rose shows the wind direction in Midtown Atlanta; color coded to wind speed in miles per hour from October 7th to October 16th. The meteorological station is situated at Bobby Dodd Stadium (33.77 °N, 84.39 °W) approximately 1 km from the Ford ES&T Building. The data is available at https://gatech.weatherstem.com/data?refer=/stadium.

Chemical structures

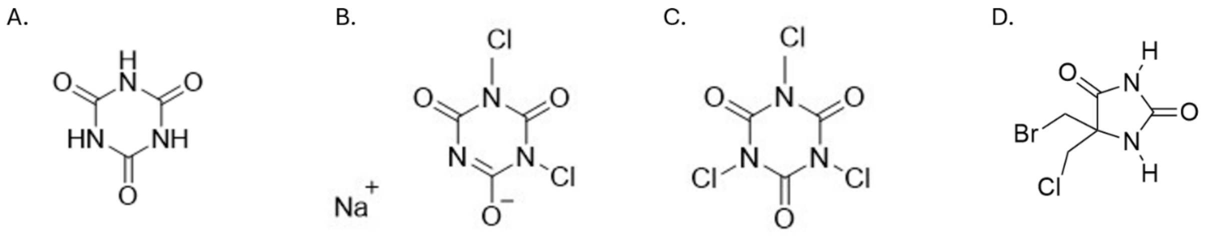

Figure S3. The structure in the leftmost panel (panel A) is cyanuric acid (CYA). The structure in panel B is sodium dichloroisocyanurate (DCCA). The structure in panel C is trichloroisocyanuric acid (TCCA). Bromochloro-5,5-dimethylimidazolidine-2,4-dione (BCDMH) is shown in the far-right panel (panel D).

Sensitivities/Calibrations

Table S1. Sensitivities of Calibrated Compounds

Species	Sensitivity (Hz/pptv)
Acetaldehyde (C_2H_4O)[a]	2.9×10^{-4}
Bromine (Br_2)[a]	1.2
Ethylene Oxide (C_2H_4O)[b]	$<2.5 \times 10^{-5}$
Isocyanic acid (HNCO)[a]	0.062
Chlorine (Cl_2)[c]	1.5
Cyanoacetic acid ($C_3H_3NO_2$)[d]	4.7×10^{-3}
Urea (CH_4N_2O)[b]	0.64
Nitryl Chloride ($ClNO_2$)[c]	1.3
Hydrogen Cyanide (HCN)[c]	0.14
Bromine Chloride (BrCl)[a]	1.4
Trichloramine (Cl_3N)[e]	5.3
Dichloramine (Cl_2NH)[e]	1.7
Cyanamide (CH_2N_2)[c]	11
Nitryl Bromide ($BrNO_2$)[c]	1.3
Hypobromous acid (HOBr)[a]	0.6
Bromine Monoxide (BrO)[c]	2.3
Cyanogen Bromide (BrCN)[c]	7.5
Hydroxyacetonitrile (C_2H_3NO)[e]	19
Methyl Isocyanate (C_2H_3NO)[b]	$<4.6 \times 10^{-6}$
Oxalic acid ($C_2H_2O_4$)[d]	3.9
Propylene Glycol ($C_3H_8O_2$)[a]	0.33
Dimedone ($C_8H_{12}O_2$)[d]	4.5×10^{-3}
Ethyl Bromide (C_2H_5Br)[a]	3.6×10^{-7}
Butyric anhydride ($C_8H_{14}O_3$)[a]	1.0×10^{-6}
Trifluoroacetic acid ($C_2HF_3O_2$)[a]	59
Chloroacetic acid ($C_2H_3ClO_2$)[c]	23
Glyoxal ($C_2H_2O_2$)[a]	5.6×10^{-11}

a- 220 ions/extraction

b- 210 ions/extraction

c- 240 ions/extraction

d- 160 ions/extraction

e- 10^6 total ions

The HR-TOF-CIMS sensitivity for all species was calibrated in this study or estimated from previous work[1-4]. Several species, such as oxalic acid and dimedone, were calibrated, however were not identified as species present in the BioLab plume, due to low correlation with HNCO. Additionally, other species were calibrated, such as methyl isocyanate, but were ruled out as species present in the plume due to low sensitivity. Further, some species were calibrated to test the sensitivity of various classes of compounds, such as halogenated carboxylic acids.

For the species that were calibrated, uncertainty well above detection limit (Table 1) was calculated by determining the uncertainty within each aspect of the calibration and measurement process, such as uncertainty in vapor pressures, temperatures, and gas flows, then adding these uncertainties in quadrature. For species with multiple isomers the uncertainty is referring to the calibrated species.

PAN calibrations for a previous field mission were conducted before the BioLab incident, raising concern for potential impacts of Br_2 wall interaction in the CIMS, as the procedure utilized the use of Br_2 permeation tubes.[5] In these experiments, the TOF-CIMS was continuously exposed to ~15 ppbv of Br_2 for 2-3 hours on multiple occasions before the BioLab incident. This had the impact of increasing the instrumental background for Br_2. However, we also found that addition of Cl_2 to the CIMS after exposure to Br_2 could lead to displacement reactions on instrument surfaces. This decreased Cl_2 signals while elevating Br_2 signals.

In order to investigate the magnitude of this effect, the TOF-CIMS was exposed to ~15 ppbv of Br_2 continuously for 24 hours. After ceasing Br_2 addition and waiting ten minutes ~5 pbbv of Cl_2 was added to the CIMS. Cl_2 levels were initially depressed but recovered to stable levels in ~5 minutes. This effect decreased rapidly with time. One hour after the Br_2 exposure addition of Cl_2 led to an immediate rise in the Cl_2 signal with no obvious depression. Further tests at later times gave similar results.

Notably, exposure of Cl_2 always led to elevation of Br_2 signal levels but this effect was less than 20% of the Cl_2 signal. Since the TOF-CIMS was not exposed to the Br_2 permeation tube for more than three days before the first BioLab plume was observed, we conclude the perturbations due to wall interactions are significantly smaller than those observed after the 24 hour exposure test.

Table S2. GTML Instruments

Instrument	Species Measured
Tapered Element Oscillating Microbalance (TEOM)[6]	$PM_{2.5}$ particulate matter mass
Purple Air sensors	Particulate matter (0.3-10 μm), temperature, pressure, and humidity
Quadrupole CIMS	Br_2, Cl_2

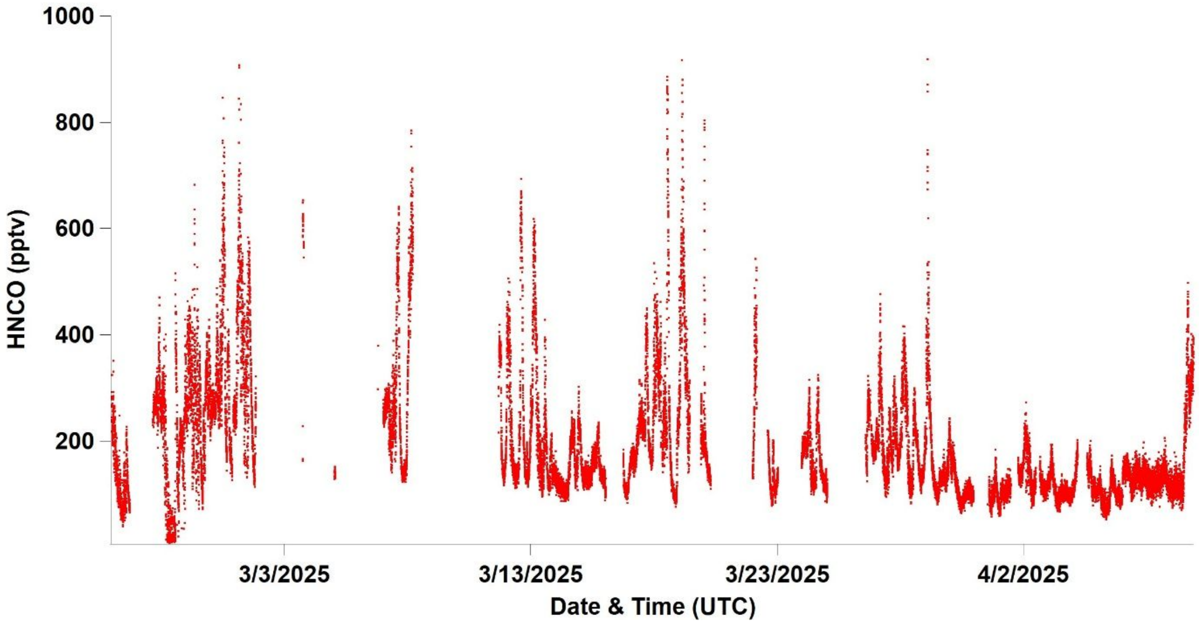

Figure S4. HNCO Time series during spring 2025 (02/23- 04/08) at South Dekalb Environmental Protection Department (EPD) Monitoring Station (33°41'16"N 84°17'25"W) approximately 13 miles southeast from metropolitan Atlanta, measured using GT I^--CIMS

Late winter and early spring are the most active seasons for controlled burns in Georgia. During this time in 2025, HNCO concentrations did not reach above 2 ppbv, unlike during BioLab plumes, supporting the use of HNCO as a BioLab tracer as background levels are almost always below 1 ppbv.

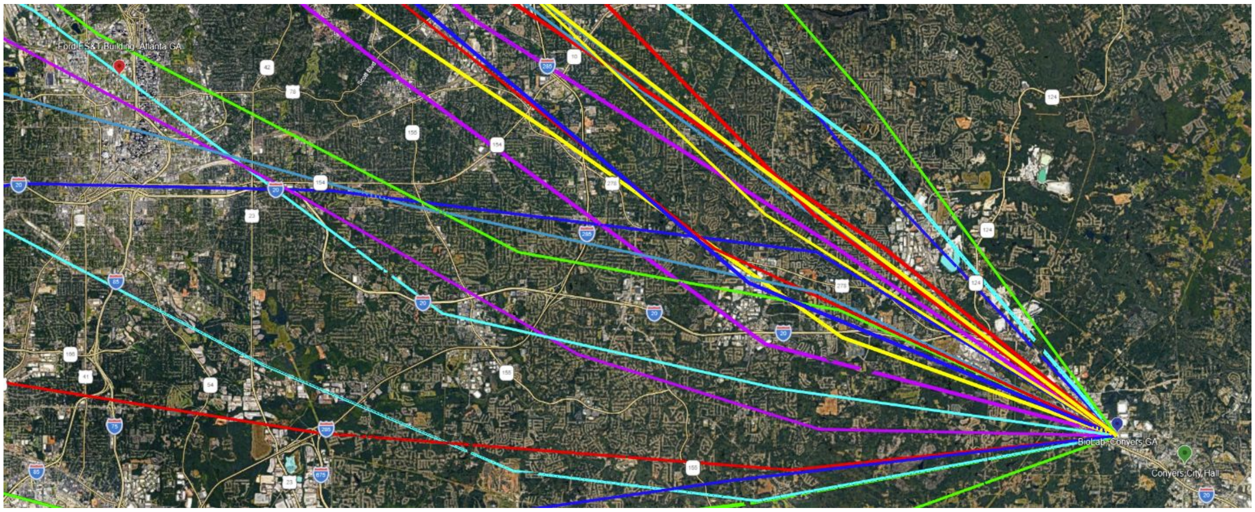

Figure S5. HYSPLIT (https://www.ready.noaa.gov/HYSPLIT.php) forward trajectories on 10/03/24 starting at 09:00 UTC originating at BioLab. Each colored line represents a trajectory at a different hour. Ford ES&T is labeled using a red marker, BioLab is labeled with a blue marker, and Conyers City Hall is labeled with a green marker.

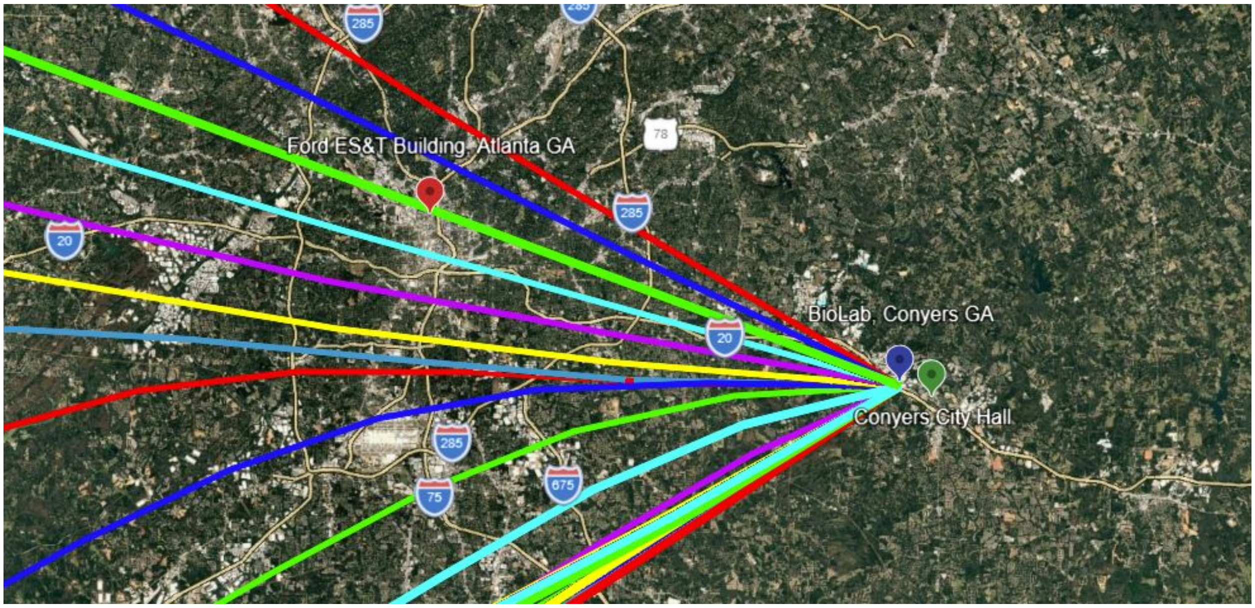

Figure S6. HYSPLIT (https://www.ready.noaa.gov/HYSPLIT.php) forward trajectories on 10/04/24 starting at 00:00 UTC originating at BioLab. Each colored line represents a trajectory at a different hour. Ford ES&T is labeled using a red marker, BioLab is labeled with a blue marker, and Conyers City Hall is labeled with a green marker.

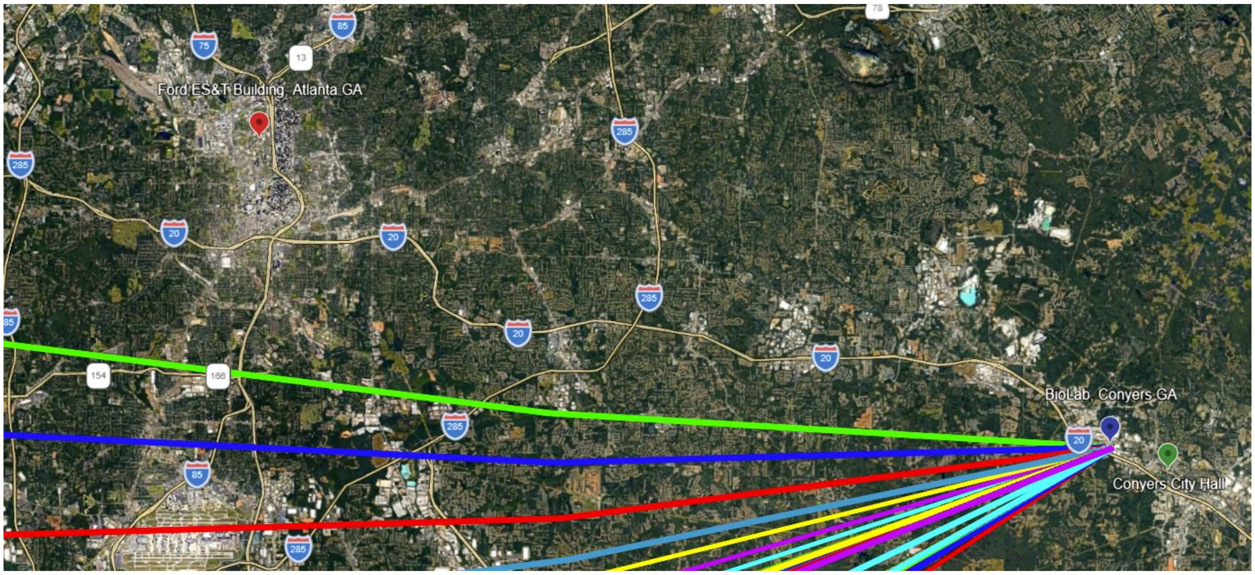

Figure S7. HYSPLIT (https://www.ready.noaa.gov/HYSPLIT.php) forward trajectories on 10/05/24 starting at 00:00 UTC originating at BioLab. Each colored line represents a trajectory at a different hour. Ford ES&T is labeled using a red marker, BioLab is labeled with a blue marker, and Conyers City Hall is labeled with a green marker.

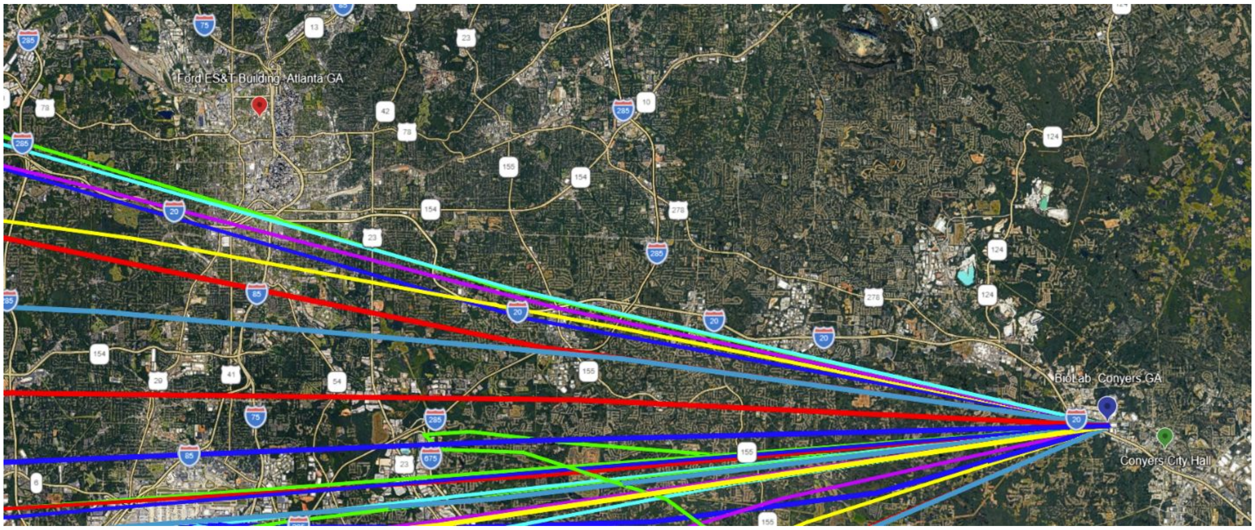

Figure S8. HYSPLIT (https://www.ready.noaa.gov/HYSPLIT.php) forward trajectories on 10/06/24 starting at 00:00 UTC originating at BioLab. Each colored line represents a trajectory at a different hour. Ford ES&T is labeled using a red marker, BioLab is labeled with a blue marker, and Conyers City Hall is labeled with a green marker.

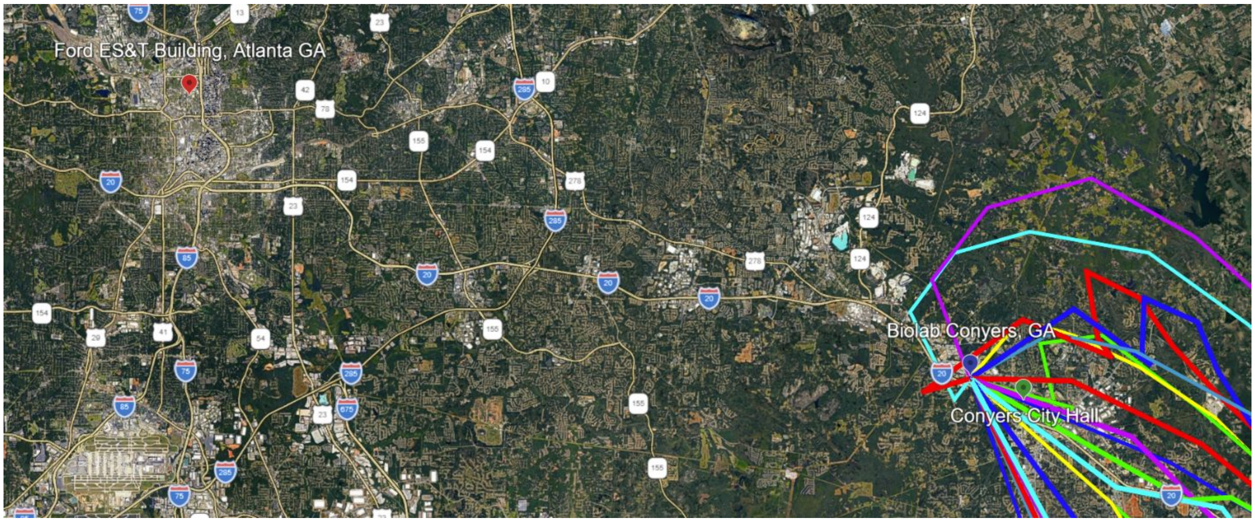

Figure S9. HYSPLIT (https://www.ready.noaa.gov/HYSPLIT.php) forward trajectories on 10/07/24 starting at 00:00 UTC originating at BioLab. Each colored line represents a trajectory at a different hour. Ford ES&T is labeled using a red marker, BioLab is labeled with a blue marker, and Conyers City Hall is labeled with a green marker.

NOAA HYPSLIT forward trajectories were made using the HRRR 3km global modeled metrological data. Every HYSPLIT modeled trajectory had a total run time of 24 hours with a new trajectory starting every hour. Default Hysplit settings were used for vertical motion and mid-boundary layer height (Model vertical velocity, no automatic mid-boundary layer height). Trajectories are modeled to be near the Ford ES&T building on 10/3, 10/4, and 10/6, with trajectories modeled further from Atlanta on 10/5 and 10/7.

Table S3. Uncalibrated Detected Species in BioLab Plume

Species	Maximum Observed Signal (ions/s)
C_2H_2O (*ketene*, ethynol, oxirene)	450
$C_2HF_3O_3$ (trifluoroperacetic acid)	450
$C_2H_2Cl_2O$ (chloroacetyl chloride, *dichloroacetaldehyde*)	130
$C_3H_6Cl_2$ (1,1-dichloropropane, 1,2-dichloropropane, 1,3-dichloropropane, 2,2-dichloropropane)	60
$C_2H_2O_2$ (glyoxal, acetylenediol, oxiranone)	50
C_2H_5N (aziridine, ethanimine, vinylamine, N-methylmethanimine)	40
$C_8H_{14}O_3$ (methyl pivaloylacetate, ethyl isobutyrylacetate)	30
C_2HNO_3 (2-nitroethenone)	17
C_2H_5ClO (*2-chloroethanol*, chloromethyl methyl ether, ethyl hypochlorite)	15

In Table S3, all the molecular formulas identified in HR-TOF-CIMS that remain uncalibrated are listed with the most likely species listed in italics.

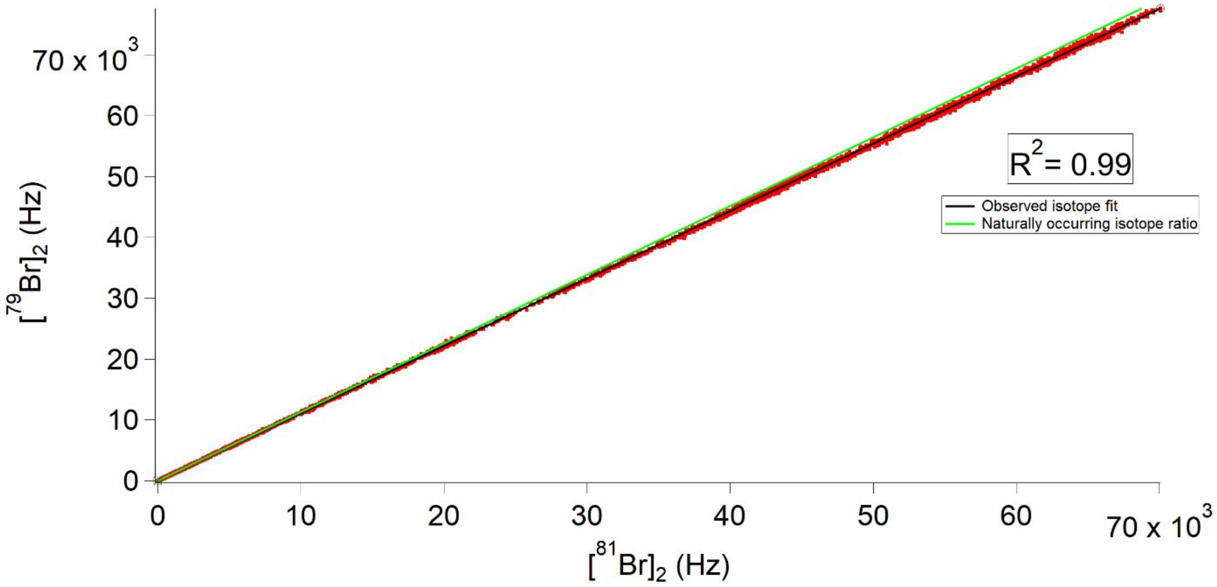

Figure S10. Bromine Isotope Correlations during the 10/03 plume in Midtown. $^{79}Br_2$ signals are on the left axis and $^{81}Br_2$ isotope signals are on the bottom axis. The naturally occurring isotope ratio is shown in green, while the observed fit line is shown in black.

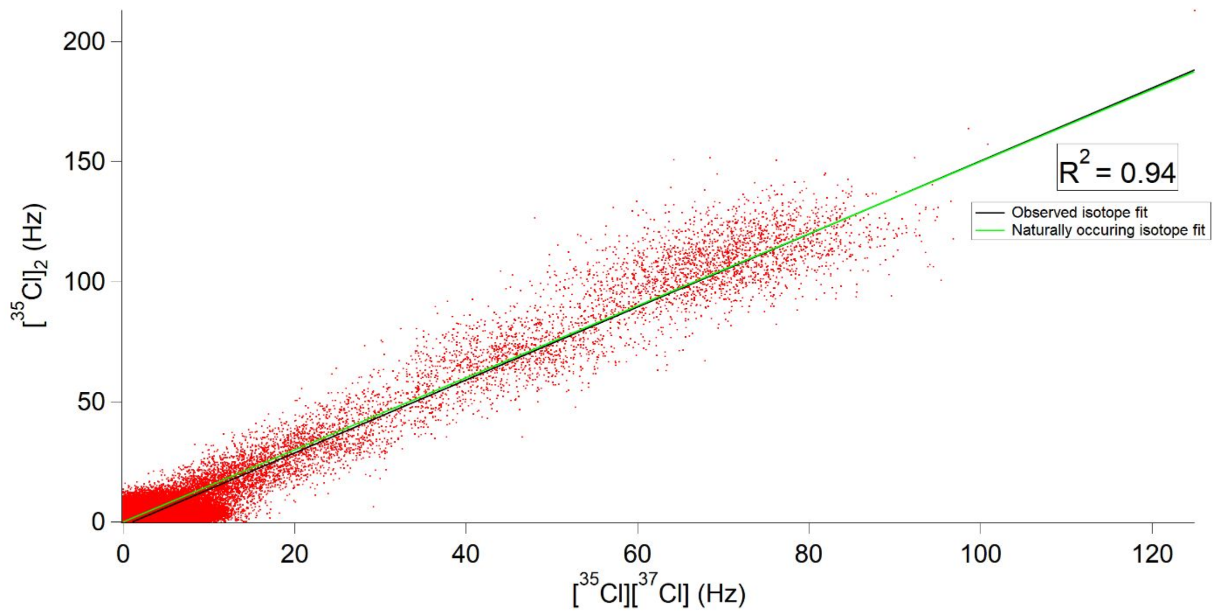

Figure S11. Chlorine isotope correlations during the 10/03 plume in Midtown. $^{35}Cl_2$ signals are on the left axis, while the $^{35}Cl^{37}Cl$ isotope signals are on the bottom axis. The naturally occurring isotope ratio is shown in green, while the observed fit line is shown in black.

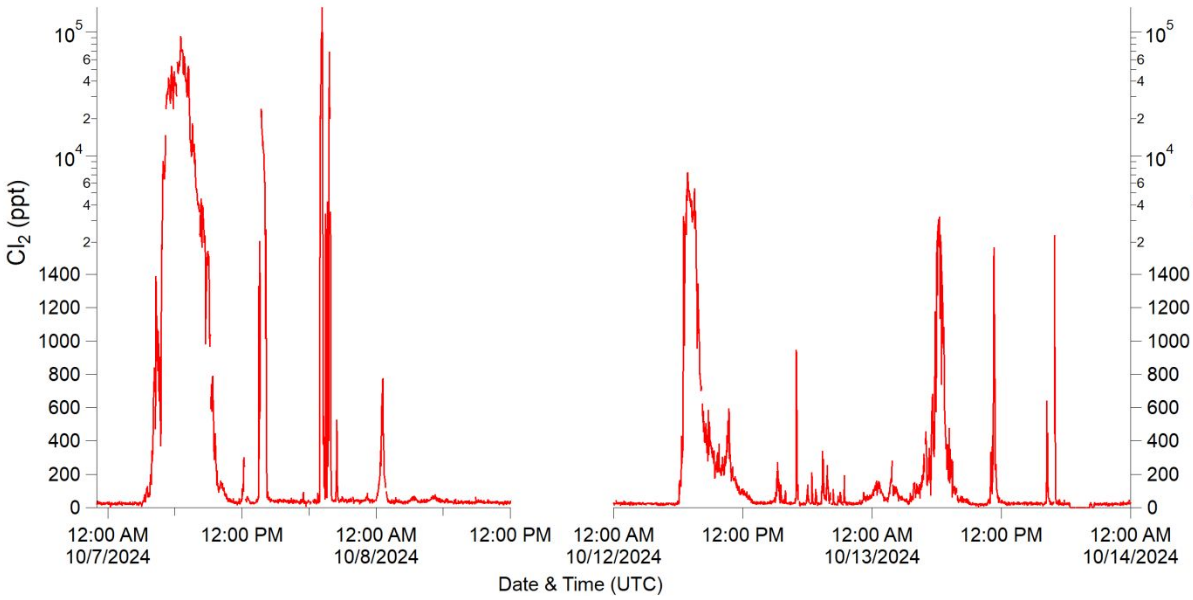

Figure S12. Time series of Cl_2 observations in Conyers GA from 10/07/2024-10/15/2024. The left axis is linear until 1400 pptv, above this level the axis is logarithmic.

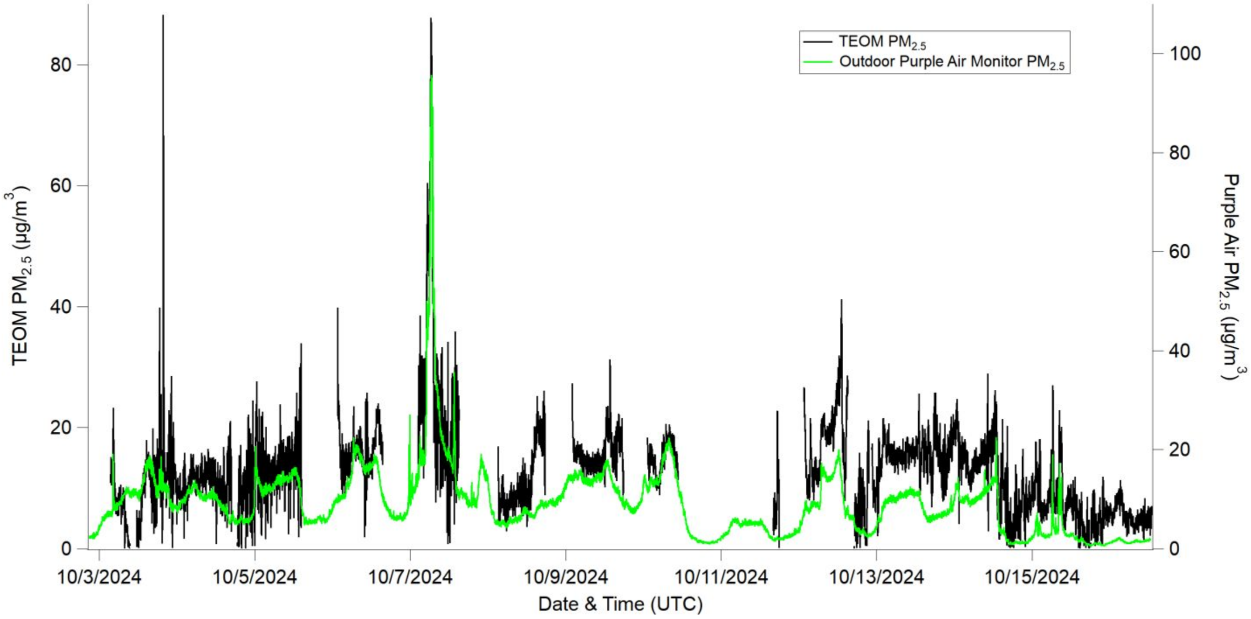

Figure S13. $PM_{2.5}$ data from the Tapered Element Oscillating Microbalance (TEOM) ambient particulate monitor (left axis, black) and $PM_{2.5}$ data from the outdoor Purple Air sensor (right axis, green) in Conyers, GA over 10/3- 10/16.

Table S4. Species Detected in BioLab Plume

Species	Highest Concentration (pptv) or Signal Observed	Calibration Method	Toxicity Notes
C_2H_4O	1.4×10^6	Acetaldehyde: ≥99.5% liquid from Sigma Aldrich made into a 8.98 ppmv gas cylinder	Acetaldehyde: NIOSH REL: Carcinogen, OHSA PEL TWA: 200 ppmv, eye, skin, and respiratory irritant[7]
Br_2	136×10^3	Permeation tube from Vici Metronics	AEGL-1: 33 ppbv<br>AEGL-2: 240 ppbv[8]
HNCO	30.4×10^3	99% solid CYA from Alfa Aesar following method described in Roberts et al 2010[9]	Exposure levels over 1.0 ppmv to be considered harmful to humans[10]
Cl_2	1.72×10^3	Permeation tube from Vici Metronics	AEGL-1: 500 ppbv<br>AEGL-2: 2000 ppbv[11]
$C_3H_3NO_2$	1.55×10^3	>98% pure solid from TCI	Severe skin and eye damage, may cause respiratory irritation, 1500 ppmv to kill 50% of rats studied[12]
CH_4N_2O	873	ACS Reagent (99.0-100.5%) solid from Sigma Aldrich	AIHA WEEL 8-hour exposure limit: 3.2 ppmv[13]
$ClNO_2$	314	Estimated using ratios between Cl_2 sensitivities in Roberts et al 2024[1]	Irritant to eyes, skin, and mucous membranes[14]
HCN	139	10 ppmv gas cylinder from GASCO	1 hr AEGL-1 threshold: 2.0 ppmv<br>1 hr AEGL-2 threshold: 7.1 ppmv[15]
BrCl	84.7	Estimated from sensitives of Br_2 and Cl_2	1 hr AEGL-2 threshold: 830 ppbv[16]
Cl_3N	42.2	Used sensitivity described in Angelucci et al 2022[2]	Lowest lethal concentration in rats over 1 hr: 112 ppmv<br>Can cause respiratory and eye irritation[2,17]

Cl_2NH	35.5	Used sensitivity described in Angelucci et al 2022[2]	Respiratory irritant[2]
CH_2N_2	27.5	98% pure solid from Beantown Chemical	NIOSH REL up to 10-hour limit: 1.16 ppmv[18]
$BrNO_2$	6.5	Assumed equivalent sensitivity to $ClNO_2$	No data found on health effects
HOBr	6.3	Estimated using ratios between Br_2 and HOBr in Liao et al 2012[4]	Skin and eye irritant[19]
BrO *	5.9	Estimated using ratios between Cl_2 sensitivities in Roberts et al 2024[1]	No data found on health effects
BrCN	1.85	Estimated using ratios between Cl_2 sensitivities in Roberts et al 2024[1]	Harms brain and heart, mucous membranes, burns skin, causes nervous system depression, chest pain and irritation[20]
C_2H_3NO	1.12	Methyl Isocyanate: 97.4% pure liquid from Chem Service Inc. made into a 8.48 ppmv gas cylinder<br>Hydroxyacetonitrile: used sensitivities described in Finewax et al 2024[21]	1 hr AEGL-2 threshold: 67 ppbv[22]
C_2H_2O	450 ions/s	N/A	Severely irritates eyes, skin, and respiratory tract[23]
$C_2HF_3O_3$	450 ions/s	N/A	No compound-specific data found on health effects. Anticipated to be similar to, however stronger than, peracetic acid due to higher acidity and oxidizing strength: corrosive to eyes and mucous membranes[24]
$C_2H_2Cl_2O$	130 ions/s	N/A	1 hr AEGL-1 threshold: 40 ppbv<br>1 hr AEGL-2 threshold: 1.6 ppmv[25]
$C_3H_6Cl_2$ *	60 ions/s	N/A	Probable carcinogen, irritant to lungs, skin, mucous membranes, listed on

			EPA list of hazardous substances[26]
$C_2H_2O_2$	50 ions/s	Glyoxal: 40% w/w aq. solution from Thermo Scientific	Skin, eye, and gastrointestinal irritation[27]
C_2H_5N	40 ions/s	N/A	Carcinogen[28]
$C_8H_{14}O_3$	30 ions/s	Butyric Anhydride: 98% pure liquid from Thermo Scientific	Depending on structure: burns skin and inflames lungs or skin and eye irritant[29,30]
C_2HNO_3	17 ions/s	N/A	Lowest lethal concentration in mice: 140.4 ppmv[31]
C_2H_5ClO *	15 ions/s	N/A	Chloromethyl methyl ether:1 hr AEGL-1 threshold: 470 ppbv[32]

*- elevated signals only during the first plume (10/03/2024)

NIOSH REL: National Institute for Occupational Safety and Health Recommended Exposure Level

OHSA PEL TWA: Occupational Safety and Health Permissible Exposure Levels is the maximum legal concentration in a workplace for a time-weighed average (TWA) period of 8 hours

AEGL-1: EPA Acute Exposure Guideline Levels where the general population would experience noticeable discomfort at the listed concentration

AEGL-2 threshold: EPA Acute Exposure Guideline Levels where irreversible or long-term health effects can take effect after one hour of exposure to listed concentrations

AIHA WEEL: American Industrial Health Association Workplace Environmental Exposure Limit

Table S5. Aerosol Ionic Composition Filters Collected in Conyers, GA

Start Time (UTC)	Stop Time (UTC)	$PM_{2.5}$ (μg/m^3)	Cl^- (μg/m^3)	Br^- (μg/m^3)	NO_3^- (μg/m^3)	$(SO_4)^{2-}$ (μg/m^3)
10/4/2024 13:00	10/5/2024 13:00	12.94	-0.0007	0.031833	0.049188	0.949155
10/6/2024 13:00	10/7/2024 13:00	26.36	0.63018	0.039336	0.35429	1.33529

Two 24-hour filter samples were obtained using a high volumetric flow rate sampler. The filters were analyzed using ion chromatography. The filter initiated on 10/6/2024 captured the large nocturnal plume and was elevated in both total mass and chloride but not bromide.

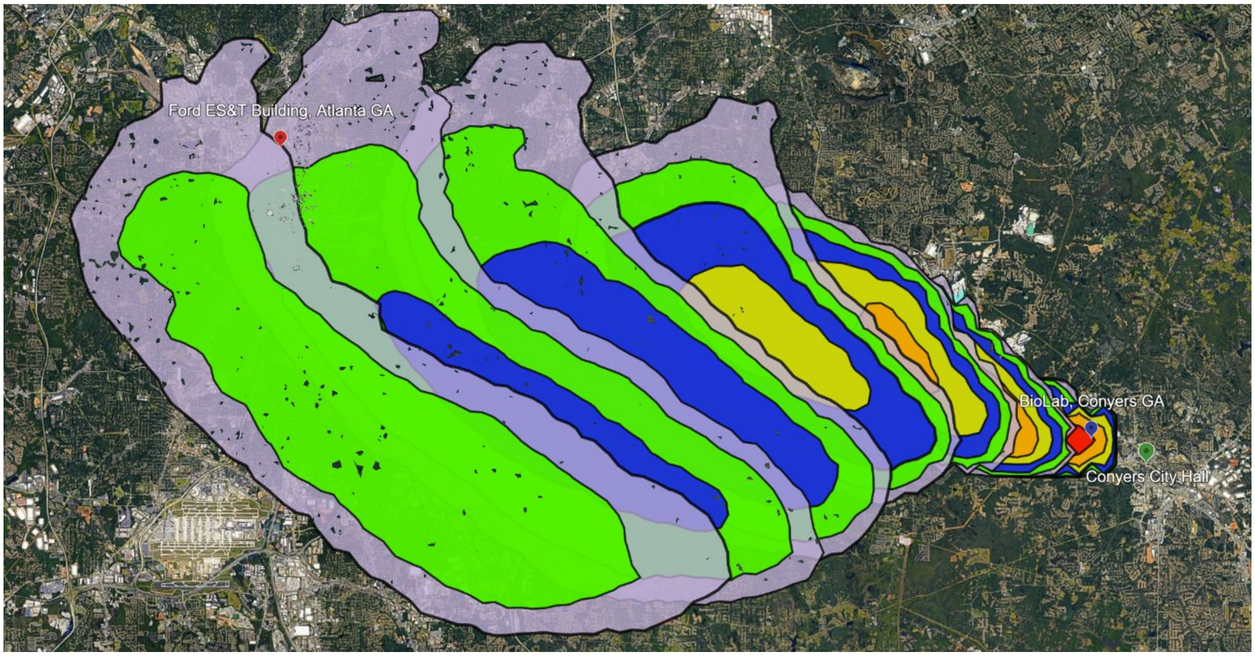

Figure S14. NOAA HYSPLIT Forward Dispersion Model (https://www.ready.noaa.gov/hypub-bin/dispasrc.pl) starting on 10/03 09:00 UTC originating at Biolab. Ford ES&T is labeled using a red marker, BioLab is labeled with a blue marker, and Conyers City Hall is labeled with a green marker. Regions are color coded based on the concentration, with red representing concentrations of 3.2 x 10^{-10} mass/m^3 or greater and purple representing concentrations of 1.0 x 10^{-12} mass/m^3. HRRR 3 km global modeled metrologic data and pollutants release type of unknown material with generic mass of duration of < 24 hours were used as inputs. Additional default settings such as, pollutant of 1 mass released for a duration of 10 minutes, no deposition, a 50 m release top, and averaging periods of 30 minutes, were used for the inputs of the model.

Using the dispersion model, the areas surrounding Biolab experience concentrations of 3.2 x 10^{-10} mass/m^3, while near Ford ES&T experience concentrations of 1.0 x 10^{-12} mass/m^{3,} approximately 5 hours after the initial release. Using these estimations, the areas near Biolab experience concentrations up to a factor of 300 higher than concentrations in metropolitan Atlanta.

Supplementary Information References

(1) Roberts, J. M.; Wang, S.; Veres, P. R.; Neuman, J. A.; Robinson, M. A.; Bourgeois, I.; Peischl, J.; Ryerson, T. B.; Thompson, C. R.; Allen, H. M.; Crounse, J. D.; Wennberg, P. O.; Hall, S. R.; Ullmann, K.; Meinardi, S.; Simpson, I. J.; Blake, D. Observations of Cyanogen Bromide (BrCN) in the Global Troposphere and Their Relation to Polar Surface O_3 Destruction. *Atmos. Chem. Phys.* **2024**, *24* (6), 3421–3443. https://doi.org/10.5194/acp-24-3421-2024.

(2) Angelucci, A. A.; Crilley, L. R.; Richardson, R.; Valkenburg, T. S. E.; Monks, P. S.; Roberts, J. M.; Sommariva, R.; VandenBoer, T. C. Elevated Levels of Chloramines and Chlorine Detected near an Indoor Sports Complex. *Environ. Sci.: Processes Impacts* **2023**, *25* (2), 304–313. https://doi.org/10.1039/D2EM00411A.

(3) Finewax, Z.; Chattopadhyay, A.; Neuman, J. A.; Roberts, J. M.; Burkholder, J. B. Calibration of Hydroxyacetonitrile ($HOCH_2$ CN) and Methyl Isocyanate (CH_3 NCO) Isomers Using I^- Chemical Ionization Mass Spectrometry (CIMS). *Atmos. Meas. Tech.* **2024**, *17* (23), 6865–6873. https://doi.org/10.5194/amt-17-6865-2024

(4) Liao, J.; Huey, L. G.; Tanner, D. J.; Flocke, F. M.; Orlando, J. J.; Neuman, J. A.; Nowak, J. B.; Weinheimer, A. J.; Hall, S. R.; Smith, J. N.; Fried, A.; Staebler, R. M.; Wang, Y.; Koo, J. -H.; Cantrell, C. A.; Weibring, P.; Walega, J.; Knapp, D. J.; Shepson, P. B.; Stephens, C. R. Observations of Inorganic Bromine (HOBr, BrO, and Br_2) Speciation at Barrow, Alaska, in Spring 2009. *J. Geophys. Res.* **2012**, *117* (D14), 2011JD016641. https://doi.org/10.1029/2011JD016641.

(5) Roberts, J. M.; Neuman, J. A.; Brown, S. S.; Veres, P. R.; Coggon, M. M.; Stockwell, C. E.; Warneke, C.; Peischl, J.; Robinson, M. A. Furoyl Peroxynitrate (Fur-PAN), a Product of VOC–NO_x Photochemistry from Biomass Burning Emissions: Photochemical Synthesis, Calibration, Chemical Characterization, and First Atmospheric Observations. *Environ. Sci.: Atmos.* **2022**, *2* (5), 1087–1100. https://doi.org/10.1039/D2EA00068G.

(6) El Asmar, R.; Li, Z.; Tanner, D. J.; Hu, Y.; O'Neill, S.; Huey, L. G.; Odman, M. T.; Weber, R. J. A Multi-Site Passive Approach to Studying the Emissions and Evolution of Smoke from Prescribed Fires. *Atmos. Chem. Phys.* **2024**, *24* (22), 12749–12773. https://doi.org/10.5194/acp-24-12749-2024

(7) CDC. *Acetaldehyde- The National Institute for Occupational Safety and Health (NIOSH).* Centers for Disease Control and Prevention. https://www.cdc.gov/niosh/idlh/75070.html (accessed 2026-02-10).

(8) US EPA, O. *Bromine Results - AEGL Program*. https://www.epa.gov/aegl/bromine-results-aegl-program (accessed 2026-02-10).

(9) Roberts, J. M.; Veres, P.; Warneke, C.; Neuman, J. A.; Washenfelder, R. A.; Brown, S. S.; Baasandorj, M.; Burkholder, J. B.; Burling, I. R.; Johnson, T. J.; Yokelson, R. J.; De Gouw, J. Measurement of HONO, HNCO, and Other Inorganic Acids by Negative-Ion Proton-Transfer Chemical-Ionization Mass Spectrometry (NI-PT-CIMS): Application to Biomass Burning Emissions. *Atmos. Meas. Tech.* **2010**, *3* (4), 981–990. https://doi.org/10.5194/amt-3-981-2010.

(10) Roberts, J. M.; Veres, P. R.; Cochran, A. K.; Warneke, C.; Burling, I. R.; Yokelson, R. J.; Lerner, B.; Gilman, J. B.; Kuster, W. C.; Fall, R.; De Gouw, J. Isocyanic Acid in the Atmosphere and Its Possible Link to Smoke-Related Health Effects. *Proc. Natl. Acad. Sci. U.S.A.* **2011**, *108* (22), 8966–8971. https://doi.org/10.1073/pnas.1103352108.

(11) US EPA, O. *Chlorine Results - AEGL Program*. https://www.epa.gov/aegl/chlorine-results-aegl-program (accessed 2026-02-10).

(12) PubChem. *Cyanoacetic acid*. https://pubchem.ncbi.nlm.nih.gov/compound/9740 (accessed 2026-02-10).

(13) *UREA*. https://www2.atmos.umd.edu/~russ/MSDS/urea (accessed 2026-02-10).

(14) *Cas 13444-90-1,nitryl chloride | lookchem*. https://www.lookchem.com/casno13444-90-1.html#sds (accessed 2026-02-10).

(15) US EPA, O. *Hydrogen cyanide Results - AEGL Program*. https://www.epa.gov/aegl/hydrogen-cyanide-results-aegl-program (accessed 2026-02-10).

(16) US EPA, O. *Bromine chloride Results - AEGL Program*. https://www.epa.gov/aegl/bromine-chloride-results-aegl-program (accessed 2026-02-10).

(17) PubChem. *Nitrogen trichloride*. https://pubchem.ncbi.nlm.nih.gov/compound/61437 (accessed 2026-02-10).

(18) OSHA, *Cyanamide | Occupational Safety and Health Administration.* https://www.osha.gov/chemicaldata/371 (accessed 2026-02-10).

(19) *Hypobromous Acid Test Reagents*. Enviro Tech Chemical Services Inc.: Modesto, CA, December 05, 2025. https://envirotech.com/wp-content/uploads/2016/01/Hypobromous-Acid-Testkit-MSDS.pdf (accessed 2026-02-10)

(20) PubChem. *Cyanogen Bromide*. https://pubchem.ncbi.nlm.nih.gov/compound/10476 (accessed 2026-02-10).

(21) Finewax, Z.; Chattopadhyay, A.; Neuman, J. A.; Roberts, J. M.; Burkholder, J. B. Calibration of Hydroxyacetonitrile ($HOCH_2CN$) and Methyl Isocyanate (CH_3NCO) Isomers Using I^- Chemical Ionization Mass Spectrometry (CIMS). *Atmos. Meas. Tech.* **2024**, *17* (23), 6865–6873. https://doi.org/10.5194/amt-17-6865-2024.

(22) US EPA, O. *Methyl isocyanate Results - AEGL Program*. https://www.epa.gov/aegl/methyl-isocyanate-results-aegl-program (accessed 2026-02-10).

(23) PubChem. *Ketene*. https://pubchem.ncbi.nlm.nih.gov/compound/10038 (accessed 2026-02-10).

(24) National Research Council (US) Committee on Acute Exposure Guideline Levels. Acute Exposure Guideline Levels for Selected Airborne Chemicals: Volume 8. Washington (DC): National Academies Press (US); 2010. 7, Peracetic Acid Acute Exposure Guideline Levels. Available from: https://www.ncbi.nlm.nih.gov/books/NBK220001/ (accessed 2026-02-10).

(25) US EPA, O. *Chloroacetyl chloride Results - AEGL Program*. https://www.epa.gov/aegl/chloroacetyl-chloride-results-aegl-program (accessed 2026-02-10).

(26) PubChem. *1,2-Dichloropropane*. https://pubchem.ncbi.nlm.nih.gov/compound/6564 (accessed 2026-02-10).

(27) PubChem. *Glyoxal*. https://pubchem.ncbi.nlm.nih.gov/compound/7860 (accessed 2026-02-10).

(28) PubChem. *Aziridine*. https://pubchem.ncbi.nlm.nih.gov/compound/9033 (accessed 2026-02-10).

(29) PubChem. *Isobutyl acetoacetate*. https://pubchem.ncbi.nlm.nih.gov/compound/522677 (accessed 2026-02-10).

(30) PubChem. *Butyric anhydride*. https://pubchem.ncbi.nlm.nih.gov/compound/7798 (accessed 2026-02-10).

(31) PubChem. *Propene, 1-nitro-*. https://pubchem.ncbi.nlm.nih.gov/compound/637919 (accessed 2026-02-10).

(32) US EPA, O. *Chloromethyl methyl ether Results - AEGL Program*. https://www.epa.gov/aegl/chloromethyl-methyl-ether-results-aegl-program (accessed 2026-02-10).
